# Establishment of a bi-layered tissue engineered conjunctiva using a 3D-printed melt electrowritten poly-(ε-caprolactone) scaffold

**DOI:** 10.1007/s10792-022-02418-y

**Published:** 2022-08-06

**Authors:** Jiajun Xie, Qi Gao, Zelmira Nuñez del Prado, Nandini Venkateswaran, Hazem M. Mousa, Enrique Salero, Juan Ye, Elena M. De Juan-Pardo, Alfonso L. Sabater, Victor L. Perez

**Affiliations:** 1 Department of Ophthalmology, Ocular Surface Center, Bascom Palmer Eye Institute, University of Miami Miller School of Medicine, Miami, FL USA; 2 Interdisciplinary Stem Cell Institute, University of Miami Miller School of Medicine, Miami, FL USA; 3 Department of Ophthalmology, Second Affiliated Hospital, Zhejiang University School of Medicine, Hangzhou, China; 4 Massachusetts Eye and Ear Infirmary, Harvard Medical School, Boston, MA USA; 5 Department of Ophthalmology, Foster Center for Ocular Immunology, Distinguished Stephen and Frances Foster Chair in Ocular Immunology, Duke Eye Center, 2351 Erwin Road, Durham, NC 27705 USA; 6 Institute for Health and Biomedical Innovation (IHBI), Queensland University of Technology (QUT), Brisbane, QLD Australia

**Keywords:** Melt electrowriting, Ocular surface, Ophthalmology, Tissue engineering

## Abstract

**Purpose:**

To utilize melt electrowriting (MEW) technology using poly-(ε-caprolactone) (PCL) coupled with a 2-step co-culturing strategy for the development of a conjunctival bi-layer synthetic construct.

**Methods:**

Melt electrowritten scaffolds using PCL were fabricated using an in-house-built MEW printer. Human conjunctival stromal cells (CjSCs) and epithelial cells (CjECs) were isolated from donor tissue. A 2-step co-culture method was done by first seeding the CjSCs and culturing for 4 weeks to establish a stromal layer, followed by CjECs and co-culturing for 2 more weeks. Cultured cells were each characterized by morphology and marker expression on immunofluorescence and qPCR. The produced construct was assessed for cellular proliferation using viability assays. The bi-layer morphology was assessed using scanning electron microscopy (SEM), confocal microscopy, and immunofluorescence imaging. The expression of extracellular matrix components and TGF-b was evaluated using qPCR.

**Results:**

CjSCs were spindle-shaped and vimentin + while CjECs were polygonal and CK13 + . CjSCs showed consistent proliferation and optimal adherence with the scaffold at the 4-week culture mark. A 2-layered construct consisting of a CjSC-composed stromal layer and a CjEC-composed epithelial layer was appreciated on confocal microscopy, SEM, and immunofluorescence. CjSCs secreted collagens (types I, V, VI) but at differing amounts from natural tissue while TGF-b production was comparable.

**Conclusion:**

The 3D-printed melt electrowritten PCL scaffold paired with the 2-step co-culturing conditions of the scaffold allowed for the first approximation of a bi-layered stromal and epithelial reconstruction of the conjunctiva that can potentially improve the therapeutic arsenal in ocular surface reconstruction.

**Supplementary Information:**

The online version contains supplementary material available at 10.1007/s10792-022-02418-y.

## Introduction

The ocular conjunctiva—a thin, transparent mucous membrane consisting of a non-keratinized stratified epithelium and a vascularized connective tissue stroma—covers more ocular surface area than is apparent. Starting at the corneoscleral limbus, it extends outwards from the circumference of the limbus to cover the sclera, continues backward making a U-turn at the fornix, and comes back to line the eyelids and form the upper and lower conjunctival sacs [1, 2]. A safeguard to the integrity of the corneal epithelium, the conjunctiva enables free movement of the eye, constitutes a physical barrier to the environment, and further protects the ocular surface by secreting mucins that lubricate the tear film. The conjunctival epithelium is continuous with the corneal epithelium at the limbus and consists of 2 to 8 layers of stratified squamous cells and mucin-secreting goblet cells, while the conjunctival stroma consists of loose mesenchymal cells scattered along a matrix of collagen and glycosaminoglycans. Moreover, unlike the cornea, the conjunctival tissue is vascularized and allows for immune system surveillance of the whole ocular surface [2, 3].

Pathologies of the ocular conjunctival tissue such as autoimmune disease (Stevens-Johnson syndrome), bacterial infections (trachoma), or neoplasia, iatrogenic causes,chemical burns, trauma, or radiation exposure can lead to chronic inflammatory ocular surface conditions and conjunctival changes requiring surgical intervention. In some cases, an adequate substitute for the removed affected conjunctival tissue is required. However, in extreme cases, such as for patients with ocular cicatricial pemphigoid, Stevens-Johnson syndrome, or toxic epidermal necrolysis, complete ocular surface reconstruction might be necessary [4, 5].

Various materials have been used as a conjunctiva substitute including (i) autologous conjunctival tissue either from the affected eye or from the fellow eye [4], (ii) placenta amniotic membrane transplants [5–7], or even (iii) mucous membrane grafts from oral or nasal mucosa [8]. However, pathologies are often bilateral, precluding the ability to harvest conjunctival tissues from the fellow eye; in addition, obtaining mucous membrane grafts can prove to be technically challenging [7]. An alternative that has become increasingly relied upon in ocular surface reconstruction, as well as other procedures, are amniotic membrane transplantations (AMT) that have been used as a graft and a dressing. These AMTs have proven to be reliable and more readily available options in such patients [9, 10]. However, as with any material used for transplantation, there are reported drawbacks related to their use which include the risk of rejection and tissue retraction, particularly in patients that suffer from chronic ocular inflammation [8, 11, 12]. As such, it is heavily encouraged that alternative options are further investigated which would expand the therapeutic repertoire from which decisions can be tailored to what is best for each case.

Other biomaterials (i.e., fibrin, collagen hydrogels, gelatin, and keratin films) are used for regenerative medicine with varying degrees of success and practicality. The main drawback recognized with the use of these materials has been the lack of adequate tensile strength of the matrices [5, 13–15]. Nonetheless, fibrin (derived from human plasma) has been identified as a good scaffold to study ocular surface inflammatory diseases [13]. This has led fibrin to be used in tissue-engineering strategies including those for the conjunctiva. In a study on rabbits, conjunctival re-epithelialization was faster when the transplanted autologous conjunctiva was cultivated on autologous fibrin compared to that cultivated on an amniotic membrane or when the defect was left bare [14]. On the other hand, collagen matrices were not found to be sufficiently elastic nor transparent. The use of keratin, which is not a native component of the conjunctiva, showed superior mechanical properties but was too stiff for ocular surface reconstruction [5, 15].

In the era of emerging bio-manufacturing technologies, there is increasing interest in the development of tissue engineering strategies that could present a consistent, available, and biocompatible alternative to natural fibers for scaffold construction. An ideal tissue-engineered conjunctival construct must be thin, stable, elastic, non-immunogenic, supportive of epithelial growth, and capable of guiding tissue regeneration of the complete ocular surface in a highly organized fashion. There have been a variety of methodologies reported in the preparation of bioengineered conjunctiva substitutes with improved characteristics and reduced risk (Table 1). These techniques are each unique in their methodology with regards to scaffold used and cultured cellular components. The different bases used as a scaffold were composed of collagen [16, 17], polymers (including PCL) [18, 19, 19–21], and decellularized porcine conjunctiva [22]. In the study using decellularized porcine conjunctival tissue, transplantation of human conjunctival explants enabled generating a stable conjunctival construct [22]. Otherwise, the methodology to allow for a bilayered conjunctival construct, particularly one not requiring a full human conjunctival explant, has not been described.

**Table 1:** Literature review of methods for construction of a bioengineered conjunctival tissue substitute

Author	In vivo vs ex vivo testing	Base	Conjunctival replacement used	Conjunctival bilayer (conjunctival stromal and epithelial cells)
Zhu et al	Ex vivo	gelatin-chitosan (GC) substrate	Rabbit conjunctival epithelial cells	None
Zhou et al	Ex vivo	vitrified collagen membranes	Conjunctival epithelial cells	None
Witt et al	In vivo	porcine decellularized conjunctiva	Human conjunctival explants or human conjunctival epithelial cells	In the human conjunctival explant
Lee et al	In vivo	Modified porous PLGA matrices	Corneal epithelial cells and human stromal fibroblasts	None
Dehghani et al	In vivo	3D-printed membrane, fabricated using a gelatin, elastin and sodium hyaluronate blend	Human limbal epithelial cells	None
Drechsler et al	Ex vivo	Acellular and fibrin-containing collagen constructs	Human conjunctival epithelial cells	None
Bosworth et al	Ex vivo	electrospun scaffolds composed of PCL and decellularized tissue matrix	Human conjunctival epithelial cells	None
Ang et al	In vivo	PCL membranes	Rabbit conjunctival epithelial cells	None

Each paper described a different methodology and expands the repertoire of options at the disposal of ocular surgeons for repair of the ocular surface. Our work can add to these options by providing, to our knowledge, the first bio-engineered bi-layers conjunctival tissue substitute containing both a stromal layer composed of human conjunctival stromal cells and an epithelium composed of human conjunctival epithelial cells

Among these emerging bio-manufacturing techniques, melt electrowriting (MEW), an advanced fiber-based technology with principles related to electrospinning, has shown great promise in its ability to fabricate pre-defined architectures and tissue-engineered constructs that allow for cell attachment and proliferation which could potentially mimic organic tissue [23–25]. MEW allows, through pneumatic force and high voltage supply, the precise and continuous deposition of a thermoplastic, synthetic, molten polymer of choice at a ground collector in a pre-defined fashion determined through a linked software. This, in turn, enables the formation of highly organized and uniform scaffolds with micrometric features allowing cell growth and proliferation [23–25]. The microarchitectures possible through MEW have been implemented in a wide variety of applications across different organs [26, 27]. Scaffolds electrospun with synthetic polymers can recreate a biomimetic functional tissue with good porosity, excellent mechanical properties (strength and transparency), great material handling, and good suturability that also offers the possibility of scalable production [25]. For cornea reconstruction, Sharma et al. [28], Wilson et al. [29], and Wu et al. [30] found superior mechanical properties for poly-(ε-caprolactone) (PCL), poly-L, D-lactic acid (PLDLA), and poly-ester urethane urea (PEUU) electrospun scaffolds. Because of the qualities of these materials, there have been investigations into its use in the engineering of scaffolds for conjunctival epithelial cultivation to be used as a surgical substitute, including that of the conjunctiva [18, 31]. Nevertheless, the synthesis of a tissue-engineered bi-layered conjunctiva, containing a stromal and epithelial layer similar to that in the native conjunctiva, as a substitute for the ocular surface has proven to be particularly challenging and has not yet been achieved.

In this study, we proposed a novel technique to tissue-engineer a bi-layered conjunctiva construct with the application of MEW printing technology. PCL was selected as the synthetic biomaterial of choice due to its availability, low cost, and relative immune tolerance [18, 32, 33]. We propose a strategy for the regeneration of the conjunctival bi-layer where initial co-culturing of conjunctival stromal cells with the bioengineered synthetic scaffold provides the necessary anchor for a second layer of the conjunctival epithelium. This study combines a top-down tissue engineering approach for the conjunctival stroma construct with a first approximation of a bottom-up strategy for bi-layer conjunctival regeneration. The bi-layer architecture of this construct resembles that of the bi-layered morphology of native tissue human conjunctiva more closely than other biomaterial substitutes. This bi-layered design, in addition to the molecular and microscopic characteristics portrayed, highlight the capacity of this construct to potentially be a reliable and accessible conjunctival substitute.

## Materials and methods

### Preparation of PCL scaffolds by melt electrowriting

Melt electrowritten PCL scaffolds were fabricated using an in-house built MEW printer (Institute of Health and Biomedical Innovation, Queensland University of Technology) as previously described [23]. Briefly, poly-(ε-caprolactone) (PCL) (PC12, Corbion Purac, MW ∼83 kDa) pellets were melted at 75 C and extruded through a 23G flat-tipped metallic needle. 2.75 bar pressurized air was used to push the polymer through the needle, which was heated at 83 C and connected to a high voltage of 11 kV. The ejected electrified jet was accelerated towards a grounded flat collector located at 10.5 mm across from the needle tip. Writing with the PCL jet was achieved by connecting the jet onto the collector fixed to a motorized x–y stage, which was controlled using Mach 3 software (Newfangled Solutions LLC, Livermore Falls, Maine, USA). The programmed design consisted of interlaced diagonal lines at a translational velocity of 1.6 m s − 1, which was looped 8 times for each scaffold. Scaffolds with offset deposited fibers were prepared on an alternative in-house-built apparatus with a static collection stage under comparable conditions [23]. For this study, the fiber diameter used was 8 µm and the scaffolds were lasered-cut into 10 mm diameter discs with a thickness of 200 µm.

The scaffolds were treated with air plasma for 1 min (Harrick Plasma), sterilized with UV light for 10 min on each side, immersed in Dulbecco's Modified Eagle Medium (DMEM) (Gibco) for 2 h (h), and dried thoroughly right before seeding.

### Human conjunctival tissues

Human conjunctival tissues were collected from Sclerocorneal rims, which were obtained from the Florida Lions Eye Bank (Miami, FL). The study was in strict accordance with the tenets of the Declaration of Helsinki, and the experimental protocol was evaluated and approved by the Institutional Review Boards of the University of Miami, Miami, FL. The tissues were preserved in Optisol-GS™ (Bauch & Lomb), and the death-to-preservation time was less than 10 h. Sclerocorneal rims were collected immediately after corneal transplantation surgeries and processed no later than 12 h post-collection.

### Isolation and culture of conjunctival cells

Cell isolation and culture were performed as previously described [34]. Briefly, the conjunctival tissue was carefully dissected into pieces approximately 2 mm^2^ in size, with underlying connective tissue removed. The conjunctival tissue was then incubated with dispase II (1.2 U/mL; Invitrogen) for 1.5 h at 37 °C in 5% CO_2_ to isolate the conjunctival epithelial layer and the stromal layer.

The loosened conjunctival epithelial cells (CjECs) were collected with mechanical scraping and recovered with a pipette. The dispase II solution containing cells was centrifuged at 1200 rpm for 5 min. The recovered cells were incubated in 0.25% trypsin/EDTA (Gibco) for 3 min at 37 °C and then centrifuged, re-suspended, and cultured in a CjEC culture medium under standard conditions. The CjEC culture medium was composed of DMEM supplemented with 10 mM ROCK Inhibitor Y27632 (Sigma-Aldrich), 10% FBS, and 5000 units/mL penicillin/streptomycin.

After gentle scraping of the CjECs, the conjunctival stroma was incubated in collagenase A overnight to obtain conjunctival stromal cells (CjSCs). The CjSCs were centrifuged, re-suspended, and cultured in a CjSC culture medium under standard conditions. The CjSC culture medium was composed of DMEM supplemented with 10% FBS and 5000 units/mL penicillin/streptomycin.

The culture medium was changed 1 day after seeding and every other day thereafter until the cultures reached 70 − 80% confluence at which time they were dissociated with 0.25% trypsin/EDTA for subculture.

### Two-step three-dimensional culture on scaffolds

A 2-step co-culture method was employed to fabricate a bi-layer tissue-engineered conjunctival construct. After isolation and expansion in vitro, passage one CjSCs were seeded on pre-treated scaffolds (10 mm diameter and 200 uM thickness) in step 1 at a density of 25,000 cells per scaffold and cultured with CjSC medium for 4 weeks to form a stroma-like structure. In step 2, passage one CjECs were seeded on top of the pre-seeded scaffolds at a density of 5,000 cells per scaffold, and co-cultured with CjSCs in CjEC culture medium for 2 more weeks.

### Cell proliferation and viability

The effects of cell proliferation on scaffolds were determined with PrestoBlue® viability reagent (Invitrogen), which is a live-cell assay for real-time monitoring from which assayed cells can be subsequently recovered for further culturing or use. At Days 1, 3, and 7 after the initial seeding and every 7 days afterward, PrestoBlue® was added directly to the culture medium at 10% v/v and incubated for 30 min.

Fluorescence was measured spectrophotometrically at 560 and 590 nm using a microplate reader (SpectraMax, Molecular Devices, LLC, Sunnyvale, CA). Cell number was calculated according to the standard curve established beforehand as the manufacturer instructed.

### Scanning Electronic Microscopy (SEM)

The morphology of the cells cultured on the scaffolds was investigated by SEM. Briefly, cell cultures were fixed with paraformaldehyde (PFA), subjected to graded ethanol dehydration, rinsed with graded acetone, and replaced with hexamethyldisilazane (HMDS) as follows: 1) rinse cell cultures with PBS to remove medium and debris; then 2) 4% PFA for 10 min; then 3) PBS for 10 min, twice; then 4 − 9) 10, 25, 50, 70, 95, 100% ethanol, respectively (10 min each step); then 10 − 12) 50, 67, 100% acetone (10 min each step), and then 13 − 16) 33, 50, 67, 100% HMDS (10 min each step). The cell cultures were subsequently left to air dry, and sputter-coated with gold for scanning electron microscopy (FEI XL-30 Field Emission ESEM/SEM, Philips).

### Immunofluorescence staining

For cross-sectional observation, scaffolds with cells were fixed with 4% PFA for 2 h and immersed with 30% sucrose for dehydration at 4 C overnight. The dehydrated samples were then embedded in a plastic plate with Tissue-Tek® O.C.T. Compound (VWR). The samples were kept at –20 C for several hours and transferred to –80  C to thoroughly set. The samples were then cut into slides for subsequent procedures.

Cell cultures or scaffold slides were fixed with 4% PFA for 10 min, then permeabilized and blocked in PBS with 0.3% (v/v) Saponin and Image-iT®FX signal enhancer (LifeTechnologies) at room temperature before staining with a primary antibody overnight at 4 C (Supplementary Table 1). Samples were washed three times (15 min each) with PBS, followed by incubation with the secondary antibody (Supplementary Table 1) for 45 min at room temperature. Nuclei were counter-stained with 4, 6 Diamidino-2-phenylindole (DAPI) (LifeTechnologies). Phase and fluorescent images were taken with a Zeiss Axiovert 200 inverted microscope fitted with a Zeiss AxioCam MRm digital camera image capture system and analyzed with AxioVision 4.9.1 software (Carl Zeiss AG). The thickness of the stromal and epithelial layers of each construct was calculated using scaled cross-sectional images by averaging the respective thickness at the center of 5 randomly selected slides at day 42. Results were reported as the average layer thickness of the 3 conjunctival constructs ± the standard deviation. Laser-scanning confocal images were taken with a Leica DM6000 B confocal microscope (Leica Microsystems GmbH, Wetzlar, Germany).

### Quantitative RT-PCR

The conjunctival tissues were lysed and homogenized by using a shredding system (QIAshredder; Qiagen, Valencia, CA). Total RNA was extracted by using RNeasy Mini Kit (Qiagen) and cDNA was synthesized using a reverse transcription kit for RT-PCR (iScript™ cDNA Synthesis kit, Bio-Rad, Hercules, CA) according to the manufacturer’s protocol. Real-time quantitative (q) PCR was carried out on a PCR detection system (Bio-Rad, Hercules, CA) using SYBER® Green Supermix (Bio-Rad, Hercules, CA) with primers obtained from Harvard Primer Database (Supplementary Table 2). The relative expression level of all targets was calculated by the ∆∆CT method2 and normalized with the housekeeping gene GAPDH.

### Statistical analysis

All experiments were performed in triplicate. Tissue scaffolding was done 3 times during which 3 constructs, in total, were made (*n* = 3) and compared to normal human conjunctival tissue collected as described above (*n* = 3). The mean and standard deviation of each group was calculated and compared using single-factor analysis of variance (ANOVA) in which a probability (*P*) value less than 0.05 was considered statistically significant. Data were shown as mean ± standard deviation (SD).

## Results

### Characterization of cells

The cells in this study were first characterized by their morphology, immunofluorescence, and expression of relevant markers on qPCR.

**Fig. 1:**
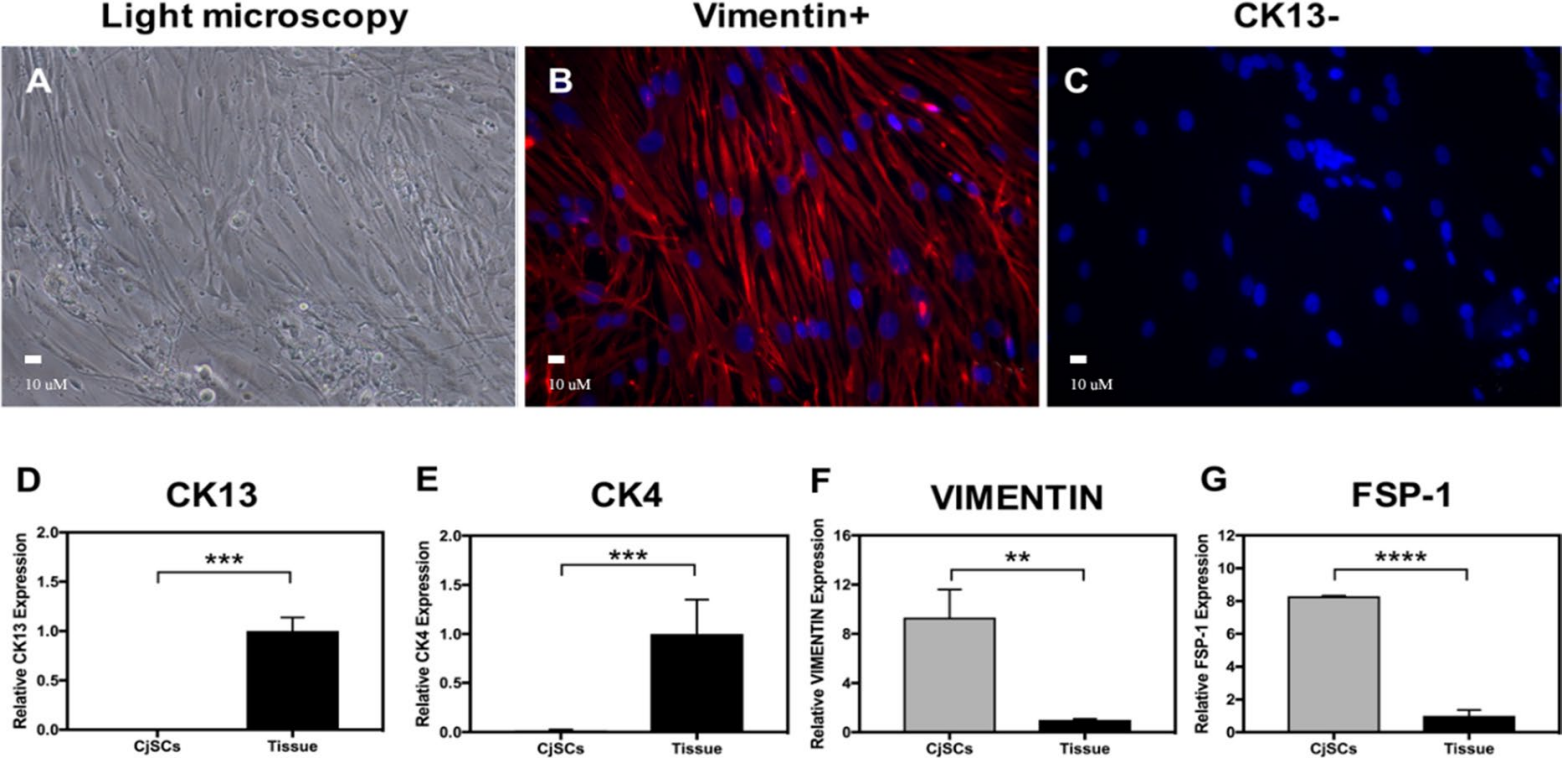
Characterization of the isolated conjunctival stromal cells (CjSCs) cultured on the melt electrowritten scaffold. The CjSCs were noted to have a spindle shape under light microscopy (**A**). Immunofluorescence (**B** and **C**) and qPCR (**D**-**G**) showed that the CjSCs cells were positive on stromal marker vimentin (red) and FSP-1 but negative on epithelial marker CK13 (green) and CK4. This highlights that the layer designed to be the conjunctival stroma was fully cultivated with CjSCs with the absence of epithelial cells. (*n* = 3/group; NS: *p* > 0.05, *: *p* ≤ 0.05; **: *p* ≤ 0.01, ***: *p* ≤ 0.001, ****p ≤ 0.0001)

Conjunctival cells were characterized by light microscopy, immunofluorescence, and qPCR (Fig. 1, Supplementary Table 1, and Supplementary Table 2). On light microscopy, CjSCs of passages 0 and 1 exhibited a spindle shape as revealed in Fig. 1A. Immunofluorescence showcased that these cells were positive for stromal markers vimentin and negative for CK13 (Fig. 1B and c). On qPCR, these cells were characterized by having significantly lower expression of the epithelial markers CK13 and CK4 (Fig. 1D and E) and significantly higher expression of stromal markers vimentin and FSP-1 compared to that of native control tissue (Fig. 1F and G).

**Fig. 2:**
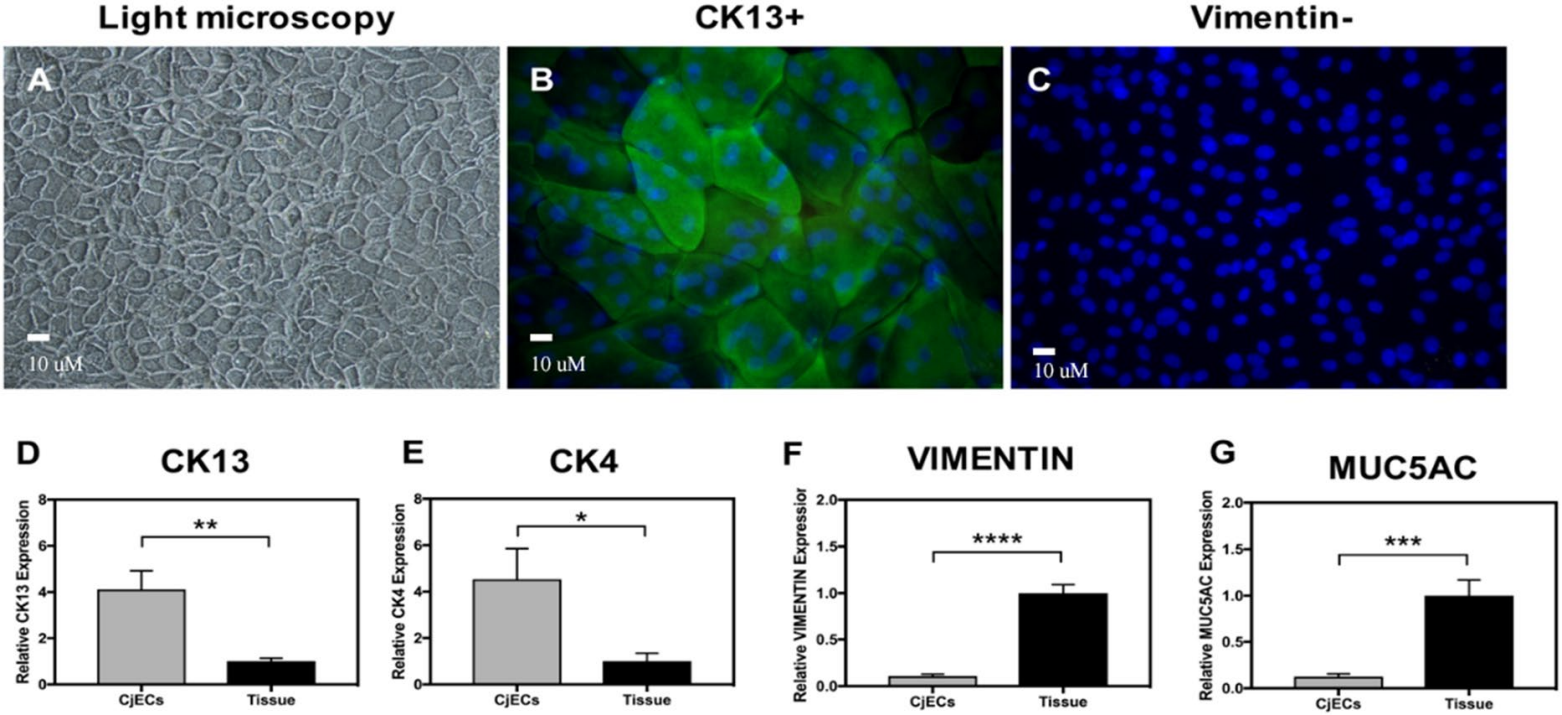
Characterization of the conjunctival epithelial cells (CjECs) in the epithelial layer of the conjunctival construct. CjECs were shown to have a polygonal shape on light microscopy (**A**). Immunofluorescence **B**-**C** showcased expression of CK13 (green) by the CjECs but negative for the stromal marker’s vimentin (red) or goblet cell marker MUC5AC. qPCR **D**-**G** highlighted expression of CK13 and CK4 by the CjECs but had reduced vimentin and MUC5AC expression compared to conjunctival tissue. (*n* = 3/group; NS: *p* > 0.05, *: *p* ≤ 0.05; **: *p* ≤ 0.01, ***: *p* ≤ 0.001, ****: *p* ≤ 0.0001)

CjECs of passages 0 and 1 appeared polygonal-shaped with distinct cell borders on light microscopy (Fig. 2A). On immunofluorescence, conjunctival epithelial marker CK4 was positively expressed in these cells, while stromal marker vimentin was negatively expressed (Fig. 2B and C). In addition, these epithelial cells where further characterized by a significantly higher expression of epithelial markers CK13 and CK4 (Fig. 2D and E) and significantly lower expression of stromal marker vimentin on qPCR (Fig. 2F) compared to that of native control tissue. Moreover, these cells did not exhibit goblet cell marker MUC5AC expression in our analysis (Fig. 2G).

### Evaluation of CjSCs proliferation and infiltration

**Fig. 3:**
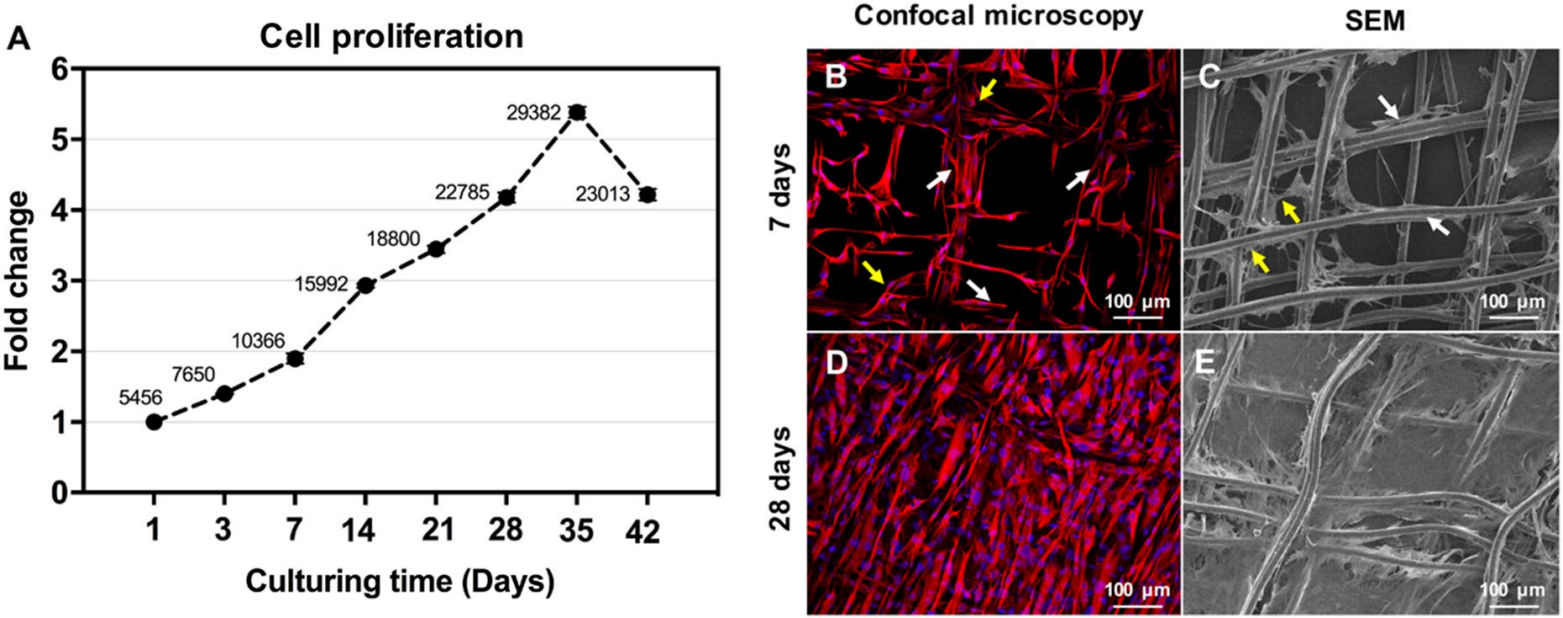
Characterization of the stromal compartment of the conjunctival construct. Passage-1 human conjunctival stromal cells’ (CjSCs) proliferation on scaffolds demonstrated a steady increase until day 35 reaching a peak at 29,382 cells highlighting the potential for cell proliferation in the scaffold (**A**). CjSCs cultured on melt electrowritten scaffolds were characterized by confocal microscopy and SEM at Day 7 (**B** and **C**, respectively) and Day 28 (**D** and **E**, respectively). CjSCs were characterized with vimentin in red and the nuclei were stained with DAPI in blue with immunofluorescence staining (**B** and **D**). CjSCs spanned along and across the fiber are indicated by white and yellow arrows, respectively

The PrestoBlue® assay was used to quantify passage 1 CjSCs proliferation on the melt electrowritten scaffolds. The data were plotted as fold-change compared with Day 1 and labeled with cell number according to the standard curve at each time point (Fig. 3A). Cell number was 5.45 ± 0.61 × 10^3^ cells on Day 1 and almost doubled by Day 7 with a cell number of 10.36 ± 0.74 × 10^3^ cells. The CjSCs proliferated within and infiltrated the melt electrowritten scaffolds during the first 5 weeks. On Day 35, cell number reached the maximum of 29.38 × 10^3^ cells and slightly declined afterward, with 23.01 ± 1.86 × 10^3^ cells on Day 42.

The morphology of CjSCs grown on the scaffolds was characterized with SEM and confocal microscopy at Days 7 and 28. After 7 days in vitro, CjSCs spanned across both the pores and fibers of the scaffold, as indicated in Fig. 3B (Confocal microscopy) and Fig. 3C (SEM) by the yellow and white arrows, respectively. After 28 days in vitro, the scaffold was completely confluent with CjSCs and the extracellular matrix (ECM) produced by the cells, as indicated in Fig. 3D (Confocal microscopy) and Fig. 3E (SEM). The CjSCs also bridged across adjacent scaffold struts, with inter-fiber distances ranging from tens to hundreds of micrometers.

### Bi-layered stromal-epithelial structure of the conjunctival construct

A two-step co-culture method was employed to establish a physiologically relevant bi-layered conjunctival construct containing both the stromal and epithelial compartments. The stromal layer was formed during step 1. Fibroblasts were allowed to grow for until complete confluency to provide a suitable stromal microenvironment for the epithelial cells. During step 2, the epithelium was generated by co-culturing CjECs cells on top of the stromal compartment for 14 days.

**Fig. 4:**
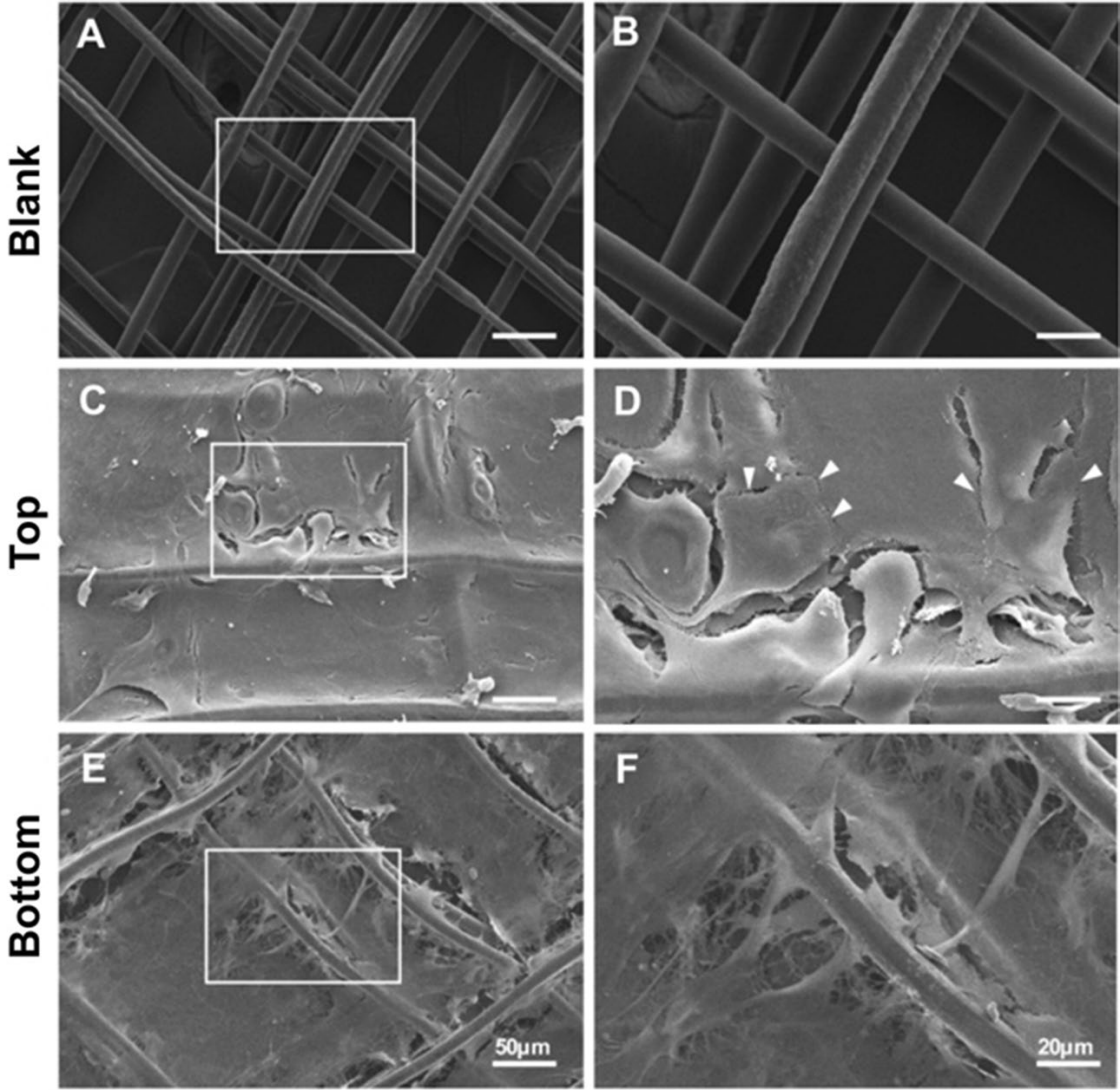
SEM images of the conjunctival construct after 2 weeks of co-culture of conjunctival stromal and epithelial cells. Blank scaffolds without cells are shown in Fig. 4A and 4B as a control. The top view (Fig. 4 C and D) and bottom view (Fig. 4E and F) of the bi-layered conjunctival construct display the conjunctival epithelial cells on top and the stroma layer beneath, respectively. Cell junctions between epithelial cells are indicated by white arrows

SEM was conducted to evaluate the bi-layered structure of the 3D conjunctival construct. SEM revealed that the elongated CjSCs and polygynous CjECs proliferated along and across the scaffold fibers in the nanoscale (Fig. 4). CjSCs and the ECM they produced filled the 3D-printed PCL as a bottom stromal layer (Fig. 4C and D), and CjECs seeded on top formed an intact epithelial layer with cell junctions indicated by white arrows (Fig. 4E and F).

**Fig. 5:**
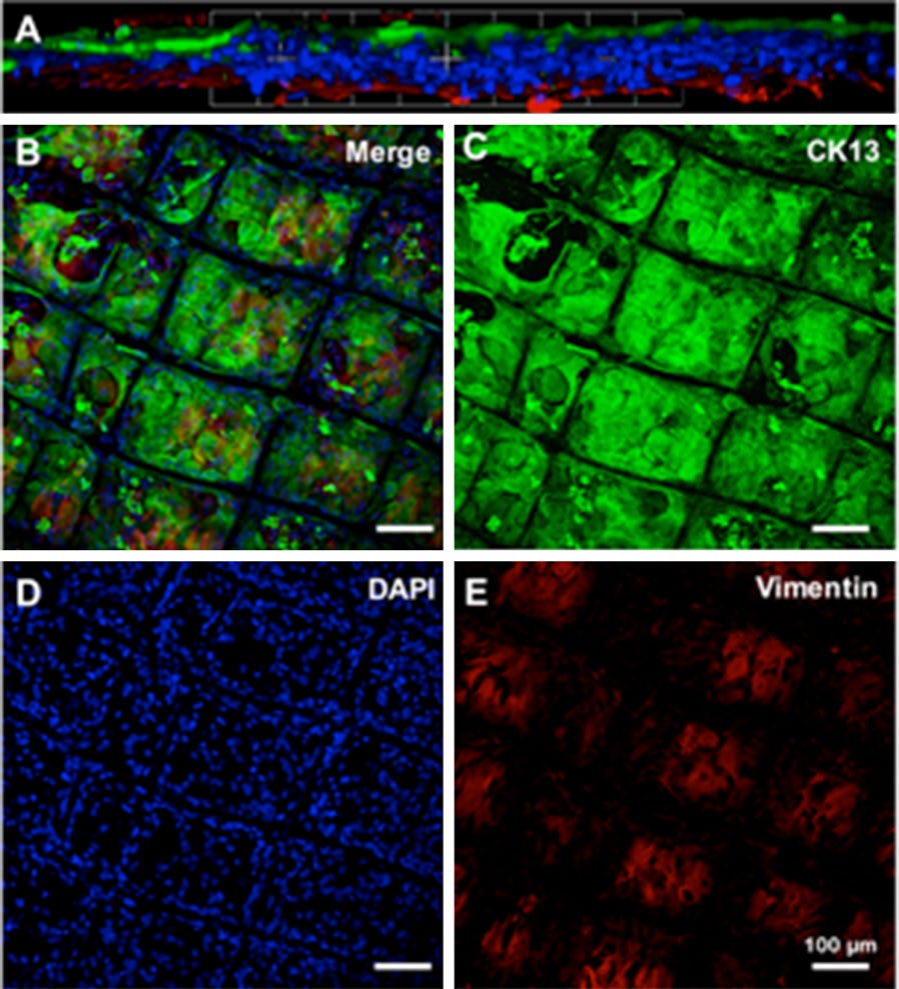
Confocal microscopy images of the conjunctival construct after being co-cultured for 2 weeks. Bi-layer conjunctival constructs were achieved on 3D-printed PCL scaffolds. 3D reconstructed images (Fig. 5A and B) showed distinct layers of CjECs (Fig. 5C) characterized with CK13 in green, and CjSCs characterized with vimentin (Fig. 5E) in red. Epithelial-to-mesenchymal transition (EMT) was not observed for conjunctival epithelial cells, nor was mesenchymal-to-epithelial transition evident for conjunctival stromal cells. Nuclei were stained with DAPI in blue (Fig. 5D)

Moreover, confocal microscopy was carried out to further visualize the bi-layer conjunctival constructs achieved on the melt electrowritten scaffolds. The 3D reconstructed confocal microscopy image (Fig. 5A and B) shows distinct layers of CjECs on top and CjSCs beneath after co-culture for 2 weeks in-vitro. CjECs were stained with CK13 in green (Fig. 5C), nuclei were stained with DAPI in blue (Fig. 5D), and CjSCs were stained with vimentin in red (Fig. 5E). This suggested that the construct and methodology allowed the formation of a bi-layer of stromal and epithelial conjunctival cells and there was no evidence of metaplasia. It was noted, however, that epithelial-to-mesenchymal transition was not observed for conjunctival epithelial cells, nor was mesenchymal-to-epithelial transition evident for conjunctival stromal cells. Epithelial cells did not lose cell polarity or intercellular adhesion (e-cadherin), while mesenchymal cells expressed the intermediate filament protein vimentin as part of their highly developed cytoskeleton structure.

**Fig. 6:**
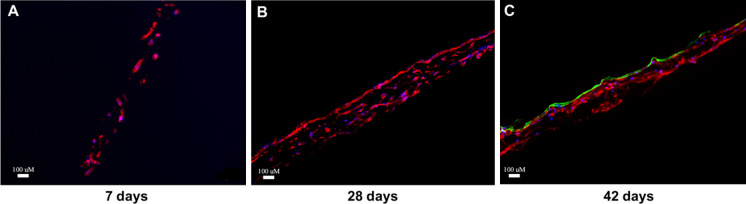
Cross-section immunofluorescence images of the conjunctival construct. CjSCs were seeded on the surface of the scaffold and presented a slow growth at Day 7 (Fig. 6A). CjSCs’ presence and infiltration throughout the scaffold were revealed with an increased number of cellular components at Day 28 (Fig. 6B). CjSCs were characterized with vimentin in red, CjECs were characterized with CK13 in green, and the nuclei were stained with DAPI in blue. A conjunctival epithelial layer was formed after 42 days of culture as shown by the high expression of CK13 staining (green) (Fig. 6C)

In addition to confocal analysis, immunofluorescence of a cross-section of the conjunctival constructs was performed to evaluate cell infiltration and matrix deposition across the full scaffold thickness. CjSCs were seeded on the surface of the scaffold and presented a slow growth at Day 7 (Fig. 6A). CjSCs’ presence and infiltration throughout the scaffold were revealed with an increased number of cellular components at Day 28 (Fig. 6B). After co-culture for 2 weeks at Day 42, an intact layer of CjECs was formed on top of the stromal layer (Fig. 6C), which further verified the SEM and confocal microscopy results. The average thickness of the stromal layer was 193.3 ± 13.4 uM and that of the epithelial layer was 38.6 ± 6.2 uM.

### Assessment of the bi-layered construct compared to native human conjunctival tissue

**Fig.7:**
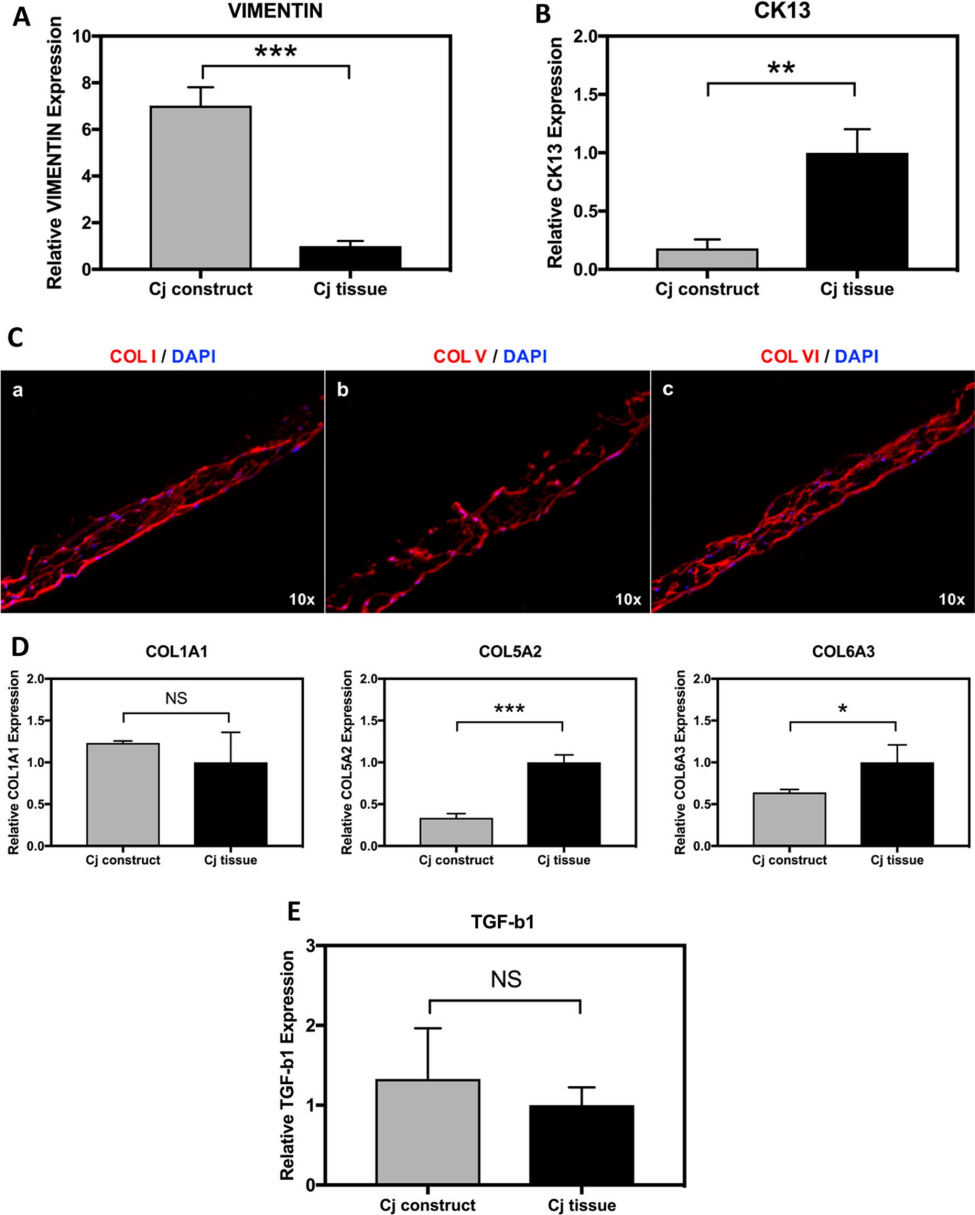
Comparison of native conjunctival tissue (Cj tissue) and the conjunctival construct. qPCR results indicated that the conjunctival construct had significantly higher vimentin expression than conjunctival tissue while having a lower expression of CK13 (7A and B). On immunofluorescence staining, CjSCs produced collagen I, V, and VI, and formed a loose and irregular ECM network. COL1A1, COL5A2, and COL6A3 expression on 3D-printed PCL scaffolds and conjunctiva tissue was evaluated by qPCR (Fig. 7C and 7D). Moreover, there was no significant difference between the conjunctival construct and tissue on TGF-b1 expression (Fig. 7E). (*n* = 3/group; NS: *p* > 0.05, *: *p* ≤ 0.05; **: *p* ≤ 0.01, ***: *p* ≤ 0.001, ****: *p* ≤ 0.0001)

The cell markers of the bi-layer conjunctival construct were examined by qPCR (Fig. 7A and B). As compared with human conjunctival tissue, the bi-layer conjunctival construct showed significantly higher expression of vimentin (*P* = 0.0002) but lower expression of CK13 (*P* = 0.0028) at the mRNA level.

To further evaluate the microenvironment created by the CjSCs during 3D culture on the scaffolds, ECM protein expression of collagen I, V, and VI was investigated by immunofluorescence staining, and mRNA levels were quantified by qPCR. As revealed in Fig. 7, the CjSCs produced abundant collagen I, collagen V, and collagen VI (Fig. 7C andD), and formed a loose and irregular ECM network. As compared with native conjunctival tissue, there were no significant differences in collagen I expression of the conjunctival construct. However, the bi-layered conjunctival construct exhibited a 3.0- and 1.5-fold decrease in collagen V and VI transcripts in comparison to native tissue, respectively.

As a key molecule of scar formation, TGF-b1 expression was investigated by qPCR (Fig. 7E). There was no statistical difference between the expression of TGF-b1 in the bi-layered conjunctival construct and the native tissue. It is noteworthy that no evidence of scaffold contraction was observed after CjSC seeding, which may reduce the possibility of scar formation of conjunctival reconstruction.

## Discussion

Fabricating three-dimensional, porous, polymer scaffolds for tissue engineering applications using additive manufacturing technology has gained rising interest. Constructed scaffolds can be combined with cells or tissue to ultimately be implanted in the human body. The scaffolds can be created with numerous additive manufacturing technologies, including the use of electrospinning technology [35–37]. With traditional solution electrospinning, voltages are applied to polymers to allow for deposition of fibers via electrostatic drawing. Polymers are typically dissolved in a solvent, and modulation of solution and processing variables can permit the deposition of fibers [36]. However, with the recent advent of MEW technology, there is increased control of several parameters for scaffold construction. Many different thermoplastic polymers can be employed for scaffold construction. The polymer used in our study was PCL, which is a slowly degrading, biocompatible, biodegradable polymer that has been widely processed by MEW in prior studies [37].

The different approaches for generating bio-engineered conjunctival substitutes reported in the literature are summarized in Table 1. Each of these techniques utilizes a different approach, base, and cellular compartment and serves to provide more options in ocular surface reconstruction for tailoring therapy and potentially improving outcomes. Nonetheless, aside from utilizing a human conjunctival explant, the ability to produce a bi-layered conjunctival construct using tissue engineering and seeded donor cells has not been reported.

This study utilizes the MEW techniques in order to approximate a tissue-engineered bi-layered conjunctival substitute. Such a product could potentially expand the therapeutic repertoire in ocular surface reconstruction. There were two parts to this study: (a) the co-culturing of conjunctival stromal cells with the engineered PCL MEW scaffold to generate a conjunctival stromal construct, and (b) the co-culturing of conjunctival epithelial cells with the engineered conjunctival stromal construct to generate a conjunctival epithelial-stromal bi-layer. These 2 parts were carried out and allowed the construct to have the distinct stromal and epithelial layers which were tested.

### Characterization and proliferation of the cultured cells

The CjSCs and CjECs were first characterized using morphology, immunofluorescence, and expression of relevant markers on qPCR. CjSCs were characterized by their spindle shape, expression of stromal marker vimentin and FSP-1, and lack of expression of epithelial markers CK13 and CK4 (Fig. 1). CjECs were characterized by their polygonal morphology, expression of epithelial markers CK13 and CK4, and lack of expression of stromal marker vimentin (Fig. 2). Overall, the cell profile for the cells indicate that each layer of the conjunctiva is composed mainly of its assigned cell type. Characterization of these cells was possible and allowed for proceeding with the experimental testing that follows. Nonetheless, some differing expressions compared to native conjunctival tissue were noted. The CjECs in our study did not exhibit MUC5AC expression indicating that differentiation into goblet cells was not achieved in our study. We posit that this absence of goblet cells is secondary to the study settings which were ex vivo and short-term in nature (Fig. 2G).

After characterization of the cells, proliferation capacity of the CjSCs of passage 1 was tested out in the construct. The PrestoBlue ® assay and demonstrated a steady increase until day 35 reaching a peak at 29.38 × 10^3^ cells highlighting the potential for cell proliferation in the scaffold (Fig. 3A). In addition, the characterized CjSCs were observed on confocal microscopy and SEM and showcased capacity to proliferate and span along and across the scaffold fibers (Fig. 3B and C). As such, the lattice-structural design with high alignment of the pore network of the scaffold and plasma-treated nanofibers succeeded in modelling the natural environment and allowed the proliferation of the CjSCs. However, day 35 to 42 exhibited a slight reduction in cell count from ~ 29 × 10^3^ to ~ 23,000 × 10^3^ (Fig. 3A). Such a decrease could be secondary to a fluctuation in the weekly cell count secondary to experimental parameters (i.e. culture settings, scaffold size); however, it could also indicate that the culturing conditions require dynamic changes to allow for a more sustained proliferation. As such, the chronic sustainability of proliferation and its implication on cell counts remains to be proven by in vivo long-term follow-up experiments.

Overall, the technique led to the production of a construct that allowed the identification and characterizing of at least 2 distinct cell populations. Conjunctival stromal and epithelial cells characterized by immunostaining with intermediate filament proteins vimentin and CK13, respectively, showed cells had retained their native cell morphology. In addition, the lattice design of the scaffold succeeded in hosting the CjSCs cells which were able to substantially proliferate in the order of weeks. Rationally, the lattice design can also serve an added advantage by allowing the infiltration of a vascular network that can allow the construct to model according to its surrounding. Nonetheless, despite demonstrating multiple aspects in which the construct resembles the natural conjunctival tissue, in the ex vivo testing mentioned, differing expressions, lack of MUC5AC expression, and fluctuations in cell count were noted.

It has been noted that the epithelial layer is difficult to grow *in-vitro*; with obstacles such as reduced survival or lack of stratification being reported in such attempts [13, 38, 39]. This difficulty is particularly true for achieving goblet cell presence. However, for goblet cells to be present in the epithelium, more than a stromal layer to grow on is required. Garcia-Posadas et al. proposed an explanation for loss of goblet cell function by suggesting that by Day 11 goblet cells had either died or had fully secreted all mucin and, therefore, were ‘invisible’ to HPA staining for MUC5AC [38]. Although the short term nature of these experiments could be a factor for the absence of goblet cells, we would like to propose that epithelial-to-goblet cell differentiation and the fate of goblet cells depend on their function, which is not tissue-generating but instead, one of defence that depends on an immune system and, therefore, cannot fulfil its purpose ex vivo. We posit that the presence of goblet cells in complex tissue assemblies is dependent on triggers by protein sensors that unless there is a trigger by protein sensors in the microenvironment and would behave differently in an in vivo setting. Goblet cell differentiation and increased formation has been noted in other studies using transplanted conjunctival cells [22, 40]. The prediction is that after implantation of a regenerated conjunctival construct, we should see differentiation of some epithelial cells into goblet cells once integration with the host system has occurred. As such, long-term in vivo follow-up experimentation which will come as a result of these pilot investigations into characterizing the methodology of the construct, could support this notion.

### Melt electrowriting 2-step co-culture method allowed the production of a bi-layered construct with a stromal and epithelial layer

SEM, confocal microscopy, and, immunofluorescence were able to demonstrate the bilayered morphology of the conjunctival construct (Figs. 4, 5, and 6). These layers were composed of a CjSC-dominant “stromal” and a CjEC-dominant epithelial layer that were identified through cellular morphology and expression profile. SEM also demonstrated that the cells were able to proliferate across the scaffold and the produced matrix. and Immunofluorescence then demonstrated chronological production of the 2 layers throughout the 2-step melt electrowriting technique in which the vimentin staining CjSCs proliferate and occupy the scaffold in the order of weeks after which the CK13-staining CjECs are seeded on top and proliferate in a distinct second “top” layer (Fig. 6). These findings highlight the successful production of a tissue-engineered conjunctival construct with distinct stromal and epithelial layers using MEW in accordance with that of a native conjunctiva. In addition, on day 42 the measurements recorded for the stromal and conjunctival layers (193 uM and 38 uM, respectively) are close to those for the native human conjunctiva reported in the literature [41]. Other techniques have been reported to produce a bi-layered conjunctival substitute. Witt et al. described a technique of utilizing decellularized porcine conjunctiva that functions as a scaffold for placement of a human conjunctival explant with epithelial cells [22]. The advantages of our methodology are the use of a synthetic scaffold and seeded conjunctival cells, both of which can be readily available and easily preserved. In scarring conditions requiring conjunctival transplantation, the availability of different options is crucial for allowing the improvement of outcomes in these patients by tailoring therapy to specific requirements.

It is also noteworthy that, on confocal microscopy, the epithelial-to-mesenchymal transition was not observed for conjunctival epithelial cells, nor was mesenchymal-to-epithelial transition evident for conjunctival stromal cells. This suggests that the morphology of the bi-layer is not entirely identical to that of the native conjunctiva. While no practical implications and characteristic differences were appreciated in the construct as a result, the implications of these findings remain to be proven in in vivo settings to observe how the construct behaves. Similar to the differences noted in cell characterization, it is likely that these alterations are related to the ex vivo culturing of the cells which does not fully resemble the natural milieu of the cells in the eye. We posit that in a more natural environment, particularly which allows vascular involvement through the porous design, these cells will behave differently and further resemble the native tissue.

### Characterization of the bi-layered construct and extracellular matrix

Epithelial cells did not lose cell polarity or intercellular adhesion (e-cadherin), while mesenchymal cells expressed the intermediate filament protein vimentin as part of their highly developed cytoskeleton structure. Nonetheless, compared to the native tissue, the conjunctival construct had higher vimentin and lower CK13 composition (Fig. 7). In addition, a variety of inferences can be made as a result. This data showcases that the cytoskeletal elements and fibers were all produced in the construct and highlight the cellular integrity; especially considering no contraction was noted in the construct. Nonetheless, these elements were expressed to a different degree compared to those in the normal conjunctiva suggesting that, in the current methods, the settings did not achieve results more comparable to native tissue. This could also suggest that the stromal-to-epithelial ratio used in these experiments was not the same as that in native tissues and may need adjusting.

The advantage of the proposed MEW technique and methodology is that it allows alterations based on the performance of the construct in order to optimize functionality and viability. It is worth noting that these changes, in the ex vivo setting, did not alter the physical characteristics of the construct in a way that could be appreciated and no contraction was encountered. The implications of these changes on the integrity and characteristics, which would drive any necessary alterations, remain to be uncovered ideally through in vivo experiments that highlight the performance of the construct in the eye and how it behaves.

All collagen types found in the native conjunctival stroma (types I, V, and VI) were also found in the cultured scaffold (Fig. 7C and D). Immunofluorescence staining, however, showed a different distribution for types of collagens (Fig. 7C and D), where non-fibrillar type collagen VI was mostly found filling in most of the porous network while collagen I (fibrillar type) appeared to overlap with the parallel alignment of conjunctival stromal cells along the mesh. Since the scaffold in this study was engineered to be strictly synthetic before being co-cultured with live cells, all genetic expression of collagens, as well as visualization of immunofluorescence, had to be uniquely produced by mature conjunctival stromal cells in the scaffold. Collagen type I was produced in abundance and to a similar extent to that in the native tissue (Fig. 7D), while collagen types V and VI were produced in quantities below those of the native tissue (Fig. 7C and D). The implications of these findings resemble those of the cytoskeletal elements (Fig. 7A and B). While these results showcase the expression of collagen types in the construct and shed light on the integrity of the produced extracellular matrix, the implications of the differing expressions compared to native tissue remain to be highlighted. No physical implications were noted in the ex vivo settings. As such, in vivo studies that test the performance of the construct are needed to demonstrate its behavior and implications of the differing composition which would allow for adjustments to the seeding protocol can optimize the composition to the desired performance.

No contraction was observed within the construct. Moreover, the qPCR analysis showed no significant difference between the constructed tissue and normal tissue in the expression of TGF-b, a key cytokine in scar formation (Fig. 7E) [42, 43]. This may suggest a low risk of scar formation and tissue contraction, a complication often seen with the use of other biomaterials [12]. In vivo experimentation can provide further confirmation on the reduced risk of scar formation with the use of such a construct.

### Limitations

This study had a variety of limitations. First, there was no histological analysis to compare the conjunctival construct with the native human conjunctival tissue and, as such, any similarities or differences in the histology or microenvironment remain to be discovered. In addition, MEW allows for extensive customizations and modifications with regards to the parameters used in the manufacturing which can alter the mechanical properties of the construct to the desired degree. As such, additional studies that determine the parameters that allow for mechanical characteristics optimal for use and resemble most those of the native tissue are required. Another major limitation was the small sample size for the comparisons between the construct and native conjunctiva. Additional studies with a larger sample size would add further validity to the findings of these tests. Moreover, the study demonstrated a new technique with the potential for allowing for a practical and accessible substitute for conjunctival tissue in cases where extractions from cadaveric donors or contralateral eyes are unavailable. However, while the results are promising, the specifics regarding how it can be adapted to meet the needs it aims to address (i.e. readily available pre-constructed tissues, compatibility with different recipients, etc.) remain to be determined. Also, the advantages of this construct compared to currently available options remain to be proven through comparative testing. Furthermore, while this study demonstrated that the scaffold allows for the adequate proliferation of the seeded conjunctival stromal cells, it did not provide chronic follow-up to highlight the long-term viability of this application and methods to prolong it; particularly when cell proliferate rate noted some fluctuations. Another major drawback was the absence of goblet cells in the construct during our testing. Such findings could be related to the ex vivo nature of the studies and we posit that the construct would behave differently in a more natural setting. As such, future in vivo studies for viability on the ocular surface need to be conducted to further characterize the utility of these conjunctival constructs. Future research should also look into the biodegradability rate of the scaffold as compared to the regeneration rate of native stromal ECM (collagens and glycosaminoglycan) so that sustained support can be guaranteed for conjunctival tissue regeneration.

### Study utility

In this pilot study, we introduce the technique of melt electrowritten PCL scaffolds seeded with conjunctival stromal and epithelial cells were engineered to serve as conjunctival constructs. The conjunctival bi-layer succeeded in preserving cell morphology and function; there was no evidence of metaplasia. Conjunctival stromal cells were able to proliferate within and infiltrate these constructs as early as 7 days, and conjunctival epithelial cells were able to survive and create a conjunctival bi-layer with the production of an ECM.

There have been a variety of bio-engineered conjunctival tissue substitutes described in the literature (Table 1). Each of these provides a new twist to the engineering methodology and materials and allowed potentially expanding the repertoire of options that can help patients across different scenarios. Compared to the other described methods for tissue-engineering for a conjunctival substitute, our proposed construct is, to our knowledge, the first of its kind in that it utilized melt electrospinning and additive manufacturing technologies for the creation of a bi-layered, composed of both human conjunctival stromal and epithelial layers, conjunctival substitute. This bi-layer is morphology is shared with that of the native conjunctiva and, pending further studies, can prove to be a useful substitute in many patients. Because of the fully cultured nature of the proposed construct, this can be readily available when needed without needing to rely on the fellow eye or cadaveric sources which may not always be feasible options. In addition, despite the present limitations, this pilot study can promote the methodology which would then allow for in vivo investigations to address the limitations.

## Conclusion

Our findings showed that the fiber composition and topography of 3D-printed PCL scaffolds, coupled with the 2 step co-culture method, allowing for the development of a bi-layered conjunctival construct with a stromal and epithelial layer. The refined lattice-structure design by the MEW method stimulated proliferation, remodelling, and adaptation of the ECM by stromal conjunctival cells. Such conditions allowed for the first approximation of a bottom-up reconstruction of the conjunctiva with a bi-layer similar to the bi-layered architecture of the native tissue. The MEW techniques used in this method are customizable and can be adapted based on additional investigations to suit the needs it aims to address. Therefore, this scaffold holds promise for an encompassing attempt to reconstruct the entire ocular surface.

## Supplementary Information

Below is the link to the electronic supplementary material.

Supplementary file1 (DOCX 13 KB)

Supplementary file2 (DOCX 13 KB)

## Data Availability

The data that support the findings of this study are available on request from the corresponding authors.

## Abbreviations

PCL: Poly-(ε-caprolactone)
PLDLA: Poly-L,D-lactic acid
PEUU: Poly-ester urethane urea
ECM: Extracellular matrix
AMT: Amniotic membrane transplantation
MEW: Melt electrowriting
DMEM: Dulbecco's modified eagle medium
CjECs: Conjunctival epithelial cells
CjSCs: Conjunctival stromal cells
PFA: Paraformaldehyde
HMDS: Hexamethyldisilazane
DAPI: Diamidino-2-phenylindole
SEM: Scanning electronic microscopy
CK13: Cytokeratin 13
EMT: Epithelial to mesenchymal transition
MET: Mesenchymal to epithelial transition
