# Supplementary material for: Establishment of a bi-layered tissue engineered conjunctiva using a 3D-printed melt electrowritten poly-(ε-caprolactone) scaffold
Source: Int Ophthalmol. 2022 Aug 6;43(1):215–32. doi: 10.1007/s10792-022-02418-y (PMC9902434; doi:10.1007/s10792-022-02418-y)
Supplement: Supplementary file 1 — Supplementary file1 (DOCX 13 KB) [file 10792_2022_2418_MOESM1_ESM.docx]

**(Supplementary) Table 1.** Antibody Sources and Concentrations

| **Antibody** | **Host and Type** | **Dilution** | **Code** | **Source** |
| --- | --- | --- | --- | --- |
| CK13 | Rabbit mo. | 1:100 | Ab92551 | Abcam, Cambridge, UK |
| Vimentin | Mouse mo. | 1:100 | SC-6260 | Santa Cruz Biotechnology, Santa Cruz, CA |
| MUC5AC | Mouse mo. | 1:50 | Ab3649 | Abcam, Cambridge, UK |
| Collagen I | Mouse mo. | 1:100 | C2456 | Sigma-Aldrich |
| Collagen V | Mouse mo. | 1:200 | MAB3393 | EMD Millipore Corporation, Billerica, MA |
| Collagen VI | Mouse mo. | 1:200 | MAB1944 | EMD Millipore Corporation, Billerica, MA |
| AlexaFluor 488 anti-rabbit IgG | Goat | 1:1000 | A-11034 | Invitrogen |
| AlexaFluor 594 anti-mouse IgG1 | Goat | 1:1000 | A-11032 | Invitrogen |
