# Supplementary material for: Establishment of a bi-layered tissue engineered conjunctiva using a 3D-printed melt electrowritten poly-(ε-caprolactone) scaffold
Source: Int Ophthalmol. 2022 Aug 6;43(1):215–32. doi: 10.1007/s10792-022-02418-y (PMC9902434; doi:10.1007/s10792-022-02418-y)
Supplement: Supplementary file 2 — Supplementary file2 (DOCX 13 KB) [file 10792_2022_2418_MOESM2_ESM.docx]

**(Supplementary) Table 2.** Primer sequences

| **Gene** | **Primers** |  |
| --- | --- | --- |
| CK4 | F | 5’-CGCGAACAGATCAAGCTCCT-3’ |
|  | R | 5’-GGGGCTCAAGGTTTTTGCTG-3’ |
| CK13 | F | 5’-GACCGCCACCATTGAAAACAA-3’ |
|  | R | 5’-TCCAGGTCAGTCTTAGACAGAG-3’ |
| VIMENTIN | F | 5’-GAGAACTTTGCCGTTGAAGC-3’ |
|  | R | 5’-GCTTCCTGTAGGTGGCAATC-3’ |
| MUC5AC | F | 5’-CAGCACAACCCCTGTTTCAAA-3’ |
|  | R | 5’-GCGCACAGAGGATGACAGT-3’ |
| fsp-1 | F | 5’-ACCTCTCTGTTCAGCACTTCC-3’ |
|  | R | 5’-CTGGGCTGCTTATCTGGGAAG-3’ |
| COL1A1 | F | 5’-GAACTTGTCACCCTCGTTGC-3’ |
|  | R | 5’-CAGATCACGTCATCGCACAAC-3’ |
| COL5A2 | F | 5’-GACTGTGCCGACCCTGTAAC-3’ |
|  | R | 5’-CCTGGACGACCACGTATGC-3’ |
| COL6A3 | F | 5’-GAGGGCCAAGACGAAGACATC-3’ |
|  | R | 5’-CAGATCACGTCATCGCACAAC-3’ |
| TGF-β1 | F | 5’-GGCCAGATCCTGTCCAAGC-3’ |
|  | R | 5’-GTGGGTTTCCACCATTAGCAC-3’ |
| GAPDH | F | 5’-AGGGCTGCTTTTAACTCTGGT-3’ |
|  | R | 5’-CCCCACTTGATTTTGGAGGGA-3’ |
